# Are Veterinary Costs and Socioeconomic Status Risk Factors for Companion Animal Relinquishment in the Republic of Korea?

**DOI:** 10.3390/ani13213406

**Published:** 2023-11-02

**Authors:** HyungChul Rah, Seok-Hwa Choi

**Affiliations:** 1 Research Institute of Veterinary Medicine, College of Veterinary Medicine, Chungbuk National University, Cheongju 28644, Republic of Korea; hrah@cbnu.ac.kr; 2 College of Veterinary Medicine, Chungbuk National University, Cheongju 28644, Republic of Korea

**Keywords:** companion animal relinquishment, dogs, cats, socioeconomic, veterinary cost

## Abstract

**Simple Summary:**

More than 110,000 companion animals go to shelters yearly due to abandonment in Republic of Korea. Veterinary costs have been blamed for the abandonment of these animals above the reported leading causes of the socioeconomic status of their owners, cost and behavior issues of animals, or housing restrictions, though without supporting evidence. This study examined whether veterinary costs and socioeconomic factors are, in fact, related to animal abandonment in 128 regions of Republic of Korea in 2020 and 2021 through a statistical analysis. Five predictors (two veterinary cost data and three socioeconomic factors) could explain one-third of animals abandoned in 2020. The same five predictors could also explain one-third of animals abandoned in 2021. These results can be used in future discussions about developing policies to reduce animal abandonment in Republic of Korea.

**Abstract:**

More than 110,000 companion animals are sent to shelters each year due to abandonment in Republic of Korea, and there is a need to analyze the causes of the relinquishment of animals and implement appropriate policies. Veterinary costs have been blamed for this issue in Republic of Korea above the reported leading causes of socioeconomic status of owners, cost and behavior issues of the animals, or housing restrictions. However, it is rare to find supporting evidence. In this study, we aimed to determine whether veterinary costs and socioeconomic factors are related to animal relinquishment in Republic of Korea. Multiple regression models were used to test if veterinary costs and socioeconomic indicators can account for relinquishment in 128 regions of Republic of Korea in 2020 and 2021. When five independent variables (two veterinary cost data and three socioeconomic indicators) were included, the regression model showed significance in explaining pet relinquishment in 2020, with an adjusted coefficient of determination of 0.3956. Pet relinquishment can also be explained by the same five variables for 2021, with an adjusted coefficient of determination of 0.391 with *p* < 0.0001. The findings suggest that intervention to reduce companion animal relinquishment in Republic of Korea should focus on lightening the financial burdens of owners as the socioeconomic status of a community worsens.

## 1. Introduction

More than 110,000 companion animals are sent to animal shelters each year in the Republic of Korea, including those that have been abandoned or lost by their owners and sent to shelters [1]. Despite funds approaching KRW 30 billion (22 m USD) allotted yearly to deal with abandoned or lost animals, the development and implementation of policies to prevent companion animal relinquishment and protect animals appropriately have been sluggish. There remains a need to analyze the causes of companion animal relinquishment accurately as a basis on which to create related policies and to implement appropriate laws to manage the abandoned animal issue.

Meanwhile, a report by the National Assembly Legislative Research Service on major legislative and policy issues of the 21st National Assembly, released in 2020, included a chapter entitled “Need for infrastructure to activate pet insurance”, which argued that due to “social problems such as the increased number of abandoned animals due to the burden of veterinary costs for companion animals”, a solution is needed [2]. Several previous studies of factors contributing to companion animal relinquishment often include the socioeconomic status of the owners, cost and behavior issues related to the companion animals directly, or housing restrictions as the leading causes [3,4,5,6,7,8,9,10,11,12,13]. It is rare to find studies that suggest that the veterinary cost burden is the main reason for relinquishment. However, one survey study exists in which participants note that providing low-cost medical care was the primary factor when asked if any aid could have kept their dogs at home, although the cost of care was not cited frequently as a reason for relinquishment [5]. In the present study, we aimed to identify factors contributing to companion animal relinquishment by region and provide a basis for preventing animals from being abandoned because communities where owners experience socioeconomic challenges and are socially vulnerable are at high risk of animal relinquishment [14,15]. We also wanted to determine whether veterinary costs for companion animals are related to their relinquishment and, if so, to compare these costs with socioeconomic factors that may also explain companion animal relinquishment.

## 2. Materials and Methods

Relevant public data were collected to identify the factors contributing to the relinquishment of companion animals in Republic of Korea. The dependent variable, the number of companion animal relinquishments by region based on local governments, was counted for the animals abandoned or lost and rescued by public animal shelters. The number of companion animal relinquishments in 228 local city, county, and district governments was sourced from the 2021 and 2022 reports on animal relinquishment by the Korean Animal Welfare Association (KAWA) [1,16]. Independent variables included here were veterinary costs by region and indicators of socioeconomic levels by region (e.g., the number of people receiving unemployment benefits, comprehensive income tax amounts, and local tax burden amount per resident). Veterinary cost data for dogs and cats from 1008 veterinary clinics and hospitals with two or more veterinarians (approximately 20% of all veterinary clinics and hospitals in Korea) were surveyed (online and on-site) and reported by region by the Ministry of Agriculture, Food, and Rural Affairs (MAFRA), Republic of Korea, in 2023 (data available at https://www.animalclinicfee.or.kr/, accessed on 4 August 2023). Among the reported veterinary cost data for dogs and cats, veterinary cost data specifically for dogs were used in the analysis, as more than 70% of companion animals in Republic of Korea are dogs, and more than 70% of abandoned companion animals were dogs in both 2020 and 2021 [1,16,17]. Among hospitalization fee data of the veterinary cost, hospitalization fee data for small dogs were used in the analysis because the most common dog breeds visiting primary-care veterinary clinics in Republic of Korea include six small toy breeds, such as Maltese, Toy Poodle, and Pomeranian, comprising approximately two-thirds of the total dogs that visited clinics [18]. The number of people receiving unemployment benefits in 2021 and 2022 by region was obtained from the unemployment benefit payment status data held by the Korea Employment Information Center. Comprehensive income tax amounts and the local tax burden amount per resident in 2021 and 2022 by region were collected from the Korean Statistical Information Service and the local finance integrated open systems, respectively. Among 228 local city, county, and district governments, data from 128 local governments were used in the analysis, as the datasets include matching data on dependent and independent variables (Table 1 and Table S1).

After ensuring the normality of the dependent variables, one of the prerequisites for a regression analysis, a left-skewed distribution, was found. Logarithmic transformation of the dependent variables was conducted to address this, resulting in skewness close to zero (Table 1).

Multiple regression models were developed to find relevant independent variables related to the increased number of relinquishments of companion animals and to estimate how much each variable contributes to the increase so that interventions can be devised to manage the abandoned animal issue. By using the veterinary cost data and the socioeconomic indicators in 2020 by region as independent variables, a least squares multiple regression analysis was conducted to estimate the number of relinquishments of companion animals in 2020 by region as a dependent variable, using MedCalc^®^ Statistical Software version 20.008 (MedCalc Software Ltd., Ostend, Belgium). The same analysis was also carried out to verify the finding using the veterinary cost data, the socioeconomic indicators in 2021, and the number of companion animal relinquishments in 2021.

## 3. Results

### 3.1. Selecting Independent Variables to Be Included in the Regression Model to Account for the Number of Companion Animal Relinquishments in 2020

Ten variables from the veterinary cost data on dogs were used as initial independent variables. When all ten variables of the veterinary cost were used, only one variable showed significance in explaining the number of companion animal relinquishments in 2020, this being X-ray and reading fee amounts. All ten veterinary cost variables showed a variance inflation factor (VIF) of less than 10, and there was no collinearity among these ten variables (Table 2). 

Three socioeconomic indicator variables were also used as initial independent variables, and all three in 2020 showed significance in explaining the number of companion animal relinquishments in 2020, specifically the number of people receiving unemployment benefits, the comprehensive income tax amount, and the local tax burden amount per resident. All three variables have VIF values of less than 10, indicating the absence of collinearity. 

Ten veterinary cost variables and three socioeconomic status variables were used as initial independent variables to account for the number of companion animal relinquishments in 2020. The regression model with 13 variables may explain about one-third of abandoned companion animals in 2020, with an adjusted coefficient of determination (R^2^-adjusted) of 0.3782, indicating statistical significance in explaining abandoned companion animals in 2020, with a *p*-value < 0.0001 (Table 3). Five variables in the model showed significance in explaining the number of companion animal relinquishments in 2020, specifically DHPPL vaccination amounts for dogs, X-ray and reading fee amounts for veterinary costs, and three socioeconomic indicators. When these five independent variables were included in the regression model, the adjusted coefficient of determination increased to 0.3956, with statistical significance, to explain abandoned companion animals in 2020. The regression coefficients of the five variables were 2.519 × 10^−4^, 1.878 × 10^−5^, 9.476 × 10^−6^, −1.726 × 10^−7^, and −2.009 × 10^−5^ for the local tax burden amount per resident, the number of people receiving unemployment benefits, the X-ray and reading fee amounts, comprehensive income tax amounts, and the DHPPL vaccination amounts for dogs, respectively. The local tax burden amount per resident had the highest contribution to the number of abandoned companion animals in 2020, whereas the DHPPL vaccination amounts for dogs showed the lowest contribution among the selected independent variables in the regression model to the number of companion animal relinquishments in 2020. The comprehensive income tax amounts and the DHPPL vaccination amounts for dogs negatively affected the number of abandoned companion animals in 2020, whereas three other variables had positive effects.

### 3.2. Verifying the Regression Model Using the Number of Companion Animal Relinquishments in 2021

A regression model using the number of companion animal relinquishments in 2021 was developed to verify the findings of the 2020 data. The same approach was applied to the 2021 data. When all ten veterinary cost variables were used as initial independent variables, only one showed significance in explaining the number of companion animal relinquishments in 2021: X-ray and reading fee amounts. All ten veterinary cost variables showed a VIF value of less than 10 (Table 4). All three socioeconomic indicator variables in 2021 also showed significance in explaining the number of companion animal relinquishments in 2021. All three socioeconomic indicator variables have a VIF value of less than 10.

Thirteen independent variables consisting of ten veterinary cost data variables and three socioeconomic indicator variables were used in the regression model to account for the number of companion animal relinquishments in 2021. The regression model with thirteen variables may explain the number of abandoned companion animals in 2021 with an adjusted coefficient of determination of 0.3782, which is statistically significant in explaining abandoned companion animals in 2021 with a *p*-value < 0.0001 (Table 5). The result of 2021 confirms the finding of 2020, with a similar adjusted coefficient of determination. The same five variables in the model also showed statistical significance in explaining the number of companion animal relinquishments in 2021. When these five independent variables were included in the regression model, the adjusted coefficient of determination increased to 0.391, with statistical significance, to explain the number of abandoned companion animals in 2021. The regression coefficients of the five variables were 2.76 × 10^−4^, 1.857 × 10^−5^, 7.762 × 10^−6^, −1.577 × 10^−7^, and −1.986 × 10^−5^ for the local tax burden amount per resident, the number of people receiving unemployment benefits, the X-ray and reading fee amounts, the comprehensive income tax amounts, and the DHPPL vaccination amounts for dogs, respectively. The local tax burden amount per resident showed the highest contribution to the number of abandoned companion animals in 2021, whereas the DHPPL vaccination amounts for dogs showed the lowest contribution among the selected independent variables in the regression model to the number of companion animal relinquishments in 2021. The comprehensive income tax amounts and the DHPPL vaccination amounts for dogs continued to negatively affect how many companion animals were abandoned in 2021, whereas the three other variables had positive effects. The regression model developed using the number of companion animal relinquishments in 2021 verified the findings of the 2020 data.

## 4. Discussion

There have been claims in the Republic of Korea that veterinary costs are unaffordable and that non-transparent costs with many variations may contribute to the abandonment of companion animals [2,19,20,21]. Very recently, the Korean government released some veterinary costs online based on a survey of 1008 veterinary clinics and hospitals with two or more veterinarians. In this study, we utilized a retrospective design in the research to explore the relationship among the number of companion animal relinquishments, the reported veterinary cost data, and socioeconomic indicators in 128 regions. The number of companion animal relinquishments, including those that had been abandoned or lost and sent to public animal shelters in the 128 regions, was 94,490 in 2020 and 84,122 in 2021, according to annual reports from KAWA [1,16]. Relinquished companion animals in this study are abandoned or lost by their owners and rescued by animal shelters. According to a previous report, there are no public animal shelters in Republic of Korea where pet owners can formally surrender animals except in very exceptional cases, and the only practical options for animal relinquishment are unofficially surrendering animals to private shelters with fees or surreptitiously dumping them [13]. Therefore, animals surrendered to private shelters are not included in the statistics. Socioeconomic status in the 128 regions was estimated from the number of people receiving unemployment benefits, the comprehensive income tax amounts, and the local tax burden amount per resident in 2020 and 2021, as reported by public governmental statistical services. Our findings show that two veterinary cost data and three socioeconomic indicators are statistically significant predictive variables to explain the number of companion animal relinquishments, suggesting that three socioeconomic indicators (e.g., increased tax burden, increased unemployment, and decreased income) and two veterinary cost data (e.g., X-ray and reading fee amounts and DHPPL vaccination amounts for dogs) contribute to the increase in companion animal relinquishments in the Republic of Korea.

A review study of 192 published articles investigating companion animal relinquishment reported that economic reasons ranked third, after housing issues and lack of time and responsibility, among the caretaker-related reasons for companion animal relinquishment [7]. A few studies have investigated factors affecting animal welfare or companion animal relinquishment, and some reported that socioeconomic factors are among these factors [6,9,10,11,13,22]. Three such studies used retrospective data as we did; however, the other three used survey data. Study locations include Canada, Australia, the United States, Spain, and Korea. Few studies were identified from other countries, despite the widespread nature of the relinquishment of companion animals globally [7].

A Canadian study used four factors of social vulnerability (e.g., low income, unemployment) to assess social determinants as predictors of animal surrender to shelters in British Columbia, Canada, finding that the social vulnerability factors, called the Canadian Index of Multiple Deprivation (CIMD) factors, including the ethnocultural composition, economic dependency, residential instability, and situational vulnerability, predicted increased risk of animal surrender to shelters across many shelters [6], which is consistent with the findings of our study. The Canadian study used geospatial analysis to identify differences in deprivation and animal relinquishment risk for various geographic areas but also cautioned that CIMD factors associated with shelter intake do not necessarily imply a causal association. Recently, a Korean study investigated the relationship between the relinquishment of dogs and cats and four open governmental socioeconomic indicators, including relocation of residence (e.g., moving), household income (e.g., unemployment), household composition (e.g., divorce), and time availability (e.g., economically inactive population) [13]. Relationships reported in the Korean study include one between a decline in household income (e.g., an increase in people receiving unemployment benefits) and an increase in the number of abandoned or lost dogs, another between increased relocations of residences, time availability, and a decrease in the number of relinquished cats, as well as positive effects of divorce on increases in numbers of abandoned or lost dogs and cats. Our findings also confirm that increased unemployment is positively related to the increase in companion animal relinquishments. An Australian study used retrospective data from canine welfare complaints to explore the relationship between canine welfare complaints and owners’ socioeconomic status in Queensland, Australia [9]. The Australian study claimed that canine welfare complaints were more commonly reported in areas with inhabitants of low socioeconomic status. It also reported that the difference in socioeconomic levels between cases citing and not citing abandonment was slight. The Australian study argues that animal welfare issues are more common in areas of low socioeconomic status; however, it also points out that animal relinquishment has multicausal reasons and that various factors, including aggressive behaviors [7,23,24], the nature of animals, such as purebred animals [23,24], human factors, such as poor health and ethics of owners [23,25,26], the human–animal bond, such as lack of care [23,27], as well as owners’ socioeconomic status and veterinary costs, play critical roles.

One of the studies that used survey data examined risk factors for relinquishment by surveying dog owners accessing an animal shelter in a low socioeconomic region of Los Angeles in the United States to relinquish their dogs [10]. Although low income or cost have been reported as factors related to pet relinquishment [5,28], many low-income pet owners keep their pets [10]. The authors aimed to explore factors other than income that related to relinquishment. More than three-quarters of those relinquishing responded to cost as a reason for relinquishment. They most frequently cited an inability to pay for the dogs’ needs, including veterinary costs, as a change in the household that led to relinquishing the dog. Given that more than 80% of the survey participants were not aware of any services available to them, the authors argued that assessing community needs and providing community-specific alternatives to relinquishment is necessary to intervene to keep dogs as owned animals from becoming homeless animals. A survey study of public and private animal shelters in Spain was carried out to estimate the number and the main characteristics of abandoned animals and the profile of the owners who relinquished their pets to the shelters [22]. The Spanish study shows that the most common reasons for abandoning animals in 2010 included “unexpected litters”, “change of address”, and “economic issues”. In contrast, the order was changed in 2013 to “economic issues”, “unexpected litters”, and “end of hunting season”, for which the economic crisis may be reflected in the reasons for animal relinquishment. Another study analyzed questionnaires and interviews with animal relinquishers in Sunshine Coast, Australia, to understand caretakers’ socioeconomic circumstances and reduce companion animals’ surrender rates [11]. The authors believe that since the caretakers’ living situation was a critical reason for relinquishment, support and services are necessary to alter the caretakers’ decision to relinquish and should be tailored to the underpinning socioeconomic geography of different regions, especially during times of socioeconomic crisis.

In the present study, the three socioeconomic indicators of unemployment, income tax, and the local tax burden, as well as veterinary cost data, were used to assess whether a relationship exists between abandoned or lost animals and veterinary costs and to compare the effect of veterinary costs on abandoned or lost animals with the effects of socioeconomic indicators if a relationship was found.

The findings of this study indicate that socioeconomic status is one of the main contributors to abandoned or lost animals, consistent with findings in previous retrospective studies [6,9,13]. The current study’s findings indicate that two veterinary cost factors contribute to abandoned or lost animals. Considering that veterinary costs can be a financial burden, depending on the owners’ socioeconomic status, and that communities where the owners experience socioeconomic challenges and are socially vulnerable are at high risk of animal relinquishment [14,15], intervention strategies to reduce the number of companion animal relinquishments should be directed to lighten veterinary costs (e.g., providing community-specific low-cost medical care) when the socioeconomic status of a community worsens (e.g., increased tax burden, increased unemployment, and decreased income). This implication may add weight to similar implications in previous studies [5,6,10].

A few limitations were identified in this study. First, the reported veterinary cost data were from 1008 veterinary clinics and hospitals with two or more veterinarians, representing approximately 20% of all veterinary clinics and hospitals in Korea. In further studies, veterinary cost data from veterinary clinics and hospitals with one veterinarian, accounting for approximately 80% of all veterinary clinics and hospitals in Korea, can be compiled, which the Korean government is expected to report in 2024. Second, the causes of dog and cat relinquishments may differ, but due to data collection limitations by region, the data pertaining to dogs and cats were grouped into a single category; this will also be addressed in future research. Third, the adverse effect of DHPPL vaccination amounts for dogs on companion animal relinquishments was not explained though more veterinary cost data can mitigate this shortcoming. Fourth, the study period overlaps with the COVID-19 pandemic when pet adoption and abandonment increased. Although it appeared that the increased adoption rate cancelled out the number of abandoned pets [29,30,31], the impact of the COVID-19 pandemic on companion animal relinquishment in the Republic of Korea has not been properly assessed in the present study. Fifth, this study’s number of animal relinquishments includes abandoned or lost animals. A method to distinguish animals intentionally abandoned by their owners from animals lost unintentionally is needed in future studies for appropriate policy implications for abandoned animal control.

## 5. Conclusions

This study presents a summary of factors related to companion animal relinquishment in 128 regions in Korea, indicating that not only socioeconomic status (e.g., increased tax burden, increased unemployment, and decreased income) but also two veterinary cost factors (X-ray and reading fee amounts and DHPPL vaccination amounts for dogs) contribute to the increase in companion animal relinquishments in Korea. The findings suggest that an intervention strategy to reduce companion animal relinquishments in the Republic of Korea should focus on lightening the financial burden for owners of companion animals when the socioeconomic status of a community worsens (e.g., increased tax burden, increased unemployment, and decreased income).

## Figures and Tables

**Table 1:** Descriptive statistics of the variables used in the analysis.

Category	Variables	Observed Region	Mean	Standard Deviation	Lowest	Highest	Coefficient of Skewness
Dependent variables	Number of animal relinquishments in 2020	128	738.2	713.91	94	6303	4.29
	Log of number of animal relinquishments in 2020	128	2.74	0.326	2.0	3.8	0.15
	Number of animal relinquishments in 2021	128	657.2	606.28	88	5235	3.89
	Log of number of animal relinquishments in 2021	128	2.69	0.327	1.9	3.7	0.12
Veterinary costs ^1^	Initial examination fee amounts for dogs (median)	128	9658.2	2429.69	4950	20,000	
	Re-visit examination fee amounts for dogs (median)	128	7762.9	2261.44	1000	16,500	
	Consultation fee amounts (median)	128	9807.0	4448.44	0	33,000	
	Hospitalization fee amounts for small dogs (median)	128	51,110.5	18,056.76	20,000	165,000	
	DHPPL vaccination amounts for dogs (median)	128	25,402.3	3744.84	15,000	38,000	
	Rabies vaccination amounts for dogs (median)	128	24,140.6	3944.47	13,500	38,000	
	Kennel cough vaccination amounts (Bordetella vaccination) for dogs (median)	128	21,632.8	4174.58	10,000	33,000	
	Canine influenza vaccination amounts (median)	128	34,480.5	4451.58	17,500	50,000	
	Complete blood count and readings fee amounts (median)	128	35,491.4	12,756.20	22,000	150,000	
	X-ray and reading fee amounts (median)	128	35,553.8	9531.61	16,500	71,500	
Socioeconomic status	Number of people receiving unemployment benefits in 2020	128	12,415.0	8228.34	1226	41,671	
	Comprehensive income tax amounts (million KRW) in 2020	128	269,973.4	461,709.72	9294	3,775,709	
	Local tax burden amount per resident (thousand KRW) in 2020	128	551.1	358.15	148	2327	
	Number of people receiving unemployment benefits in 2021	128	12,901.6	8431.91	1275	42,567	
	Comprehensive income tax amounts (million KRW) in 2021	128	324,581.6	560,675.26	11,057	4,599,753	
	Local tax burden amount per resident (thousand KRW) in 2021	128	567.7	351.21	160	2257	

^1^ Reported in 2023 by the Ministry of Agriculture, Food, and Rural Affairs (MAFRA), Republic of Korea and available at https://www.animalclinicfee.or.kr (accessed on 4 August 2023).

**Table 2:** Initial variables included in the regression model to account for the number of companion animal relinquishments in 2020.

Category	Independent Variables	Coefficient	*p*-Value	VIF	R^2^-Adjusted	F-Statistics	Significance Level
Veterinary costs	Initial examination fee amounts for dogs (median)	−1.452 × 10^−5^	0.4186	2.522	0.1043	2.4787	0.0099 **
	Re-visit examination fee amounts for dogs (median)	5.245 × 10^−6^	0.7694	2.175			
	Consultation fee amounts (median)	−5.378 × 10^−6^	0.4909	1.600			
	Hospitalization fee amounts for small dogs (median)	−1.082 × 10^−6^	0.5231	1.242			
	DHPPL vaccination amounts for dogs (median)	−1.74 × 10^−5^	0.2369	4.011			
	Rabies vaccination amounts for dogs (median)	−5.291 × 10^−6^	0.7286	4.807			
	Kennel cough vaccination amounts (Bordetella vaccination) for dogs (median)	−3.792 × 10^−6^	0.7220	2.631			
	Canine influenza vaccination amounts (median)	9.196 × 10^−6^	0.3142	2.190			
	Complete blood count and readings fees (median)	−2.068 × 10^−7^	0.9344	1.367			
	X-ray and reading fee amounts (median)	1.149 × 10^−5^	0.0005 ***	1.242			
Socio- economic status	Number of people receiving unemployment benefits in 2020	1.73 × 10^−5^	<0.0001 ***	1.093	0.2816	17.5965	0.0001 ***
	Comprehensive income tax amounts (million KRW) in 2020	−1.932 × 10^−7^	0.0007 ***	1.097			
	Local tax burden amount per resident (thousand KRW) in 2020	3.077 × 10^−4^	<0.0001 ***	1.004			

** *p* < 0.01, *** *p* < 0.001; Dependent variable: number of animal relinquishments in 2020.

**Table 3:** Multiple regression model for animal relinquishments, veterinary costs, and socioeconomic status for 2020.

Method	Category	Independent Variables	Coefficient	*p*-Value	VIF	R^2^-Adjusted	F-Statistics	Significance Level
Enter	Veterinary costs *	Initial examination fee amounts for dogs (median)	−6.219 × 10^−6^	0.6795	2.561	0.3782	6.9412	<0.0001 ***
		Re-visit examination fee amounts for dogs (median)	1.054 × 10^−6^	0.9440	2.204			
		Consultation fee amounts (median)	−5.354 × 10^−6^	0.4107	1.601			
		Hospitalization fee amounts for small dogs (median)	3.582 × 10^−7^	0.8026	1.282			
		DHPPL vaccination amounts for dogs (median)	−2.832 × 10^−5^	0.0259 *	4.248			
		Rabies vaccination amounts for dogs (median)	1.357 × 10^−5^	0.3027	5.142			
		Kennel cough vaccination amounts (Bordetella vaccination) for dogs (median)	−8.866 × 10^−6^	0.3219	2.663			
		Canine influenza vaccination amounts (median)	5.207 × 10^−6^	0.5061	2.322			
		Complete blood count and readings fee amounts (median)	1.061 × 10^−6^	0.6148	1.385			
		X-ray and reading fee amounts (median)	9.734 × 10^−6^	0.0004 ***	1.255			
	Socio- economic status	Number of people receiving unemployment benefits in 2020	1.941 × 10^−5^	<0.0001 ***	1.220			
		Comprehensive income tax amounts (million KRW) in 2020	−1.76 × 10^−7^	0.0012 **	1.155			
		Local tax burden amount per resident (thousand KRW) in 2020	2.453 × 10^−4^	0.0009 ***	1.281			
Stepwise	Veterinary costs *	DHPPL vaccination amounts for dogs (median)	−2.009 × 10^−5^	0.0027 **	1.195	0.3956	17.6275	<0.0001 ***
		X-ray and reading fee amounts (median)	9.476 × 10^−6^	0.0001 ***	1.003			
	Socio- economic status	Number of people receiving unemployment benefits in 2020	1.878 × 10^−5^	<0.0001 ***	1.154			
		Comprehensive income tax amounts (million KRW) in 2020	−1.726 × 10^−7^	0.001 **	1.114			
		Local tax burden amount per resident (thousand KRW) in 2020	2.519 × 10^−4^	0.0002 ***	1.111			

* *p* < 0.05, ** *p* < 0.01, *** *p* < 0.001; Dependent variable: number of animal relinquishments in 2020.

**Table 4:** Initial variables included in the regression model to account for the number of companion animal relinquishments in 2021.

Category	Independent Variables	Coefficient	*p*-Value	VIF	R^2^-Adjusted	F-Statistics	Significance Level
Veterinary costs	Initial examination fee amounts for dogs (median)	−1.156 × 10^−5^	0.5249	2.522	0.08438	2.1703	0.0243 *
	Re-visit examination fee amounts for dogs (median)	2.822 × 10^−6^	0.8763	2.175			
	Consultation fee amounts (median)	−6.739 × 10^−6^	0.3948	1.600			
	Hospitalization fee amounts for small dogs (median)	−9.475 × 10^−7^	0.5811	1.242			
	DHPPL vaccination amounts for dogs (median)	−2.166 × 10^−5^	0.1471	4.011			
	Rabies vaccination amounts for dogs (median)	−5.904 × 10^−6^	0.7026	4.807			
	Kennel cough vaccination amounts (Bordetella vaccination) for dogs (median)	−2.811 × 10^−8^	0.9979	2.631			
	Canine influenza vaccination amounts (median)	1.042 × 10^−5^	0.2607	2.190			
	Complete blood count and readings fee amounts (median)	4.348 × 10^−7^	0.8646	1.367			
	X-ray and reading fee amounts (median)	9.554 × 10^−6^	0.0039 **	1.242			
Socio- economic status	Number of people receiving unemployment benefits in 2021	1.733 × 10^−5^	<0.0001 ***	1.086	0.3044	19.5244	<0.0001 ***
	Comprehensive income tax amounts (million KRW) in 2021	−1.796 × 10^−7^	0.0001 ***	1.100			
	Local tax burden amount per resident (thousand KRW) in 2021	3.348 × 10^−4^	<0.0001 ***	1.015			

* *p* < 0.05, ** *p* < 0.01, *** *p* < 0.001; Dependent variable: number of animal relinquishments in 2021.

**Table 5:** Multiple regression model for animal relinquishments, veterinary costs, and socioeconomic status for 2021.

Method	Category	Independent Variables	Coefficient	*p*-Value	VIF	R^2^-Adjusted	F-Statistics	Significance Level
Enter	Veterinary costs *	Initial examination fee amounts for dogs (median)	−3.721 × 10^−5^	0.8052	2.559	0.3782	6.9424	<0.0001 ***
		Re-visit examination fee amounts for dogs (median)	−9.366 × 10^−7^	0.9504	2.212			
		Consultation fee amounts (median)	−6.705 × 10^−6^	0.3047	1.601			
		Hospitalization fee amounts for small dogs (median)	5.585 × 10^−7^	0.6976	1.282			
		DHPPL vaccination amounts for dogs (Median)	−3.192 × 10^−5^	0.0122 *	4.214			
		Rabies vaccination amounts for dogs (median)	1.424 × 10^−5^	0.2824	5.172			
		Kennel cough vaccination amounts (Bordetella vaccination) for dogs (median)	−5.267 × 10^−6^	0.5570	2.665			
		Canine influenza vaccination amounts (median)	5.831 × 10^−6^	0.4580	2.324			
		Complete blood count and readings fee amounts (median)	1.798 × 10^−6^	0.3963	1.388			
		X-ray and reading fee amounts (median)	7.437 × 10^−6^	0.0066 **	1.258			
	Socio- economic status	Number of people receiving unemployment benefits in 2020	1.943 × 10^−5^	<0.0001 ***	1.199			
		Comprehensive income tax amounts (million KRW) in 2020	−1.601 × 10^−7^	0.0004 ***	1.171			
		Local tax burden amount per resident (thousand KRW) in 2020	2.734 × 10^−4^	0.0003 ***	1.294			
Stepwise	Veterinary costs	DHPPL vaccination amounts for dogs (median)	−1.986 × 10^−5^	0.0030 **	1.178	0.391	17.3085	<0.0001 ***
		X-ray and reading fee amounts (median)	7.762 × 10^−6^	0.0014 **	1.003			
	Socio- economic status	Number of people receiving unemployment benefits in 2020	1.857 × 10^−5^	<0.0001 ***	1.134			
		Comprehensive income tax amounts (million KRW) in 2020	−1.577 × 10^−7^	0.0003 ***	1.126			
		Local tax burden amount per resident (thousand KRW) in 2020	2.76 × 10^−4^	0.0001 ***	1.114			

* *p* < 0.05, ** *p* < 0.01, *** *p* < 0.001; Dependent variable: number of animal relinquishments in 2021.

## Data Availability

The data presented in this study are available in the Supplementary Materials.

## Supplementary Materials

The following supporting information can be downloaded at https://www.mdpi.com/article/10.3390/ani13213406/s1, Table S1: Observed regions and numbers of animal relinquishments in 2020 and 2021 used in analysis.
