# Supplementary material for: Are Veterinary Costs and Socioeconomic Status Risk Factors for Companion Animal Relinquishment in the Republic of Korea?
Source: Animals (Basel). 2023 Nov 2;13(21):3406. doi: 10.3390/ani13213406 (PMC10647300; doi:10.3390/ani13213406)
Supplement: Supplementary file 1 [file animals-13-03406-s001.zip › animals-2648353-supplementary.pdf]

Supplementary Material

Table S1. Observed Regions and Human Population, Local Tax Burden Amount Per Resident, and Numbers of Animal Relinquishments in 2020 and 2021 used in the Analysis[1].

Observed region	Human Population in 2020	Local tax burden amount per resident (thousand KRW) in 2020	Number of Animal relinquishment in 2020	Human Population in 2021	Local tax burden amount per resident (thousand KRW) in 2021	Number of Animal relinquishment in 2021
Seoul Metropolitan City: 25 of 25 local governments						
Seoul_1	533,042	691	242	529,102	837	137
Seoul_2	462,664	284	378	460,067	303	374
Seoul_3	299,182	253	298	293,660	291	239
Seoul_4	574,315	247	252	569,166	271	164
Seoul_5	485,699	194	693	486,752	221	597
Seoul_6	339,996	273	184	337,416	322	153
Seoul_7	396,754	249	147	395,315	280	191
Seoul_8	230,811	398	177	229,642	453	131
Seoul_9	510,956	179	249	503,734	206	182
Seoul_10	317,366	240	242	311,694	281	156
Seoul_11	337,400	263	264	336,644	315	206
Seoul_12	385,483	255	260	380,596	294	230
Seoul_13	368,905	370	341	364,638	414	315
Seoul_14	304,819	306	128	306,337	355	139
Seoul_15	412,279	626	146	404,325	736	178
Seoul_16	285,990	376	163	281,000	442	152
Seoul_17	430,528	224	197	430,397	239	234
Seoul_18	658,338	325	246	658,801	388	196
Seoul_19	447,302	243	325	440,881	285	262
Seoul_20	376,837	431	140	375,675	471	164
Seoul_21	222,953	599	256	218,650	647	264
Seoul_22	473,307	199	261	466,746	225	276
Seoul_23	144,683	832	209	141,379	910	179
Seoul_24	122,499	1330	94	120,437	1509	88
Seoul_25	533,042	206	251	385,318	244	219
Busan Metropolitan City: 12 of 16 local governments						
Busan_1	142,918	940	769	143,207	953	764
Busan_2	228,049	211	502	221,256	229	475
Busan_3	177,125	806	503	178,614	788	482
Busan_4	262,069	227	428	256,333	271	328
Busan_5	266,866	225	392	273,226	240	347
Busan_6	353,159	258	619	355,917	272	545
Busan_7	283,211	153	490	278,857	160	455

[1] The numbers of animal relinquishments of observed regions were obtained from the 2021 and 2022 annual reports on animal relinquishment by the Korean Animal Welfare Association.

Table S1. Observed Regions and Human Population, Local Tax Burden Amount Per Resident, and Numbers of Animal Relinquishments in 2020 and 2021 used in the Analysis[1] (continued).

Observed region	Human Population in 2020	Local tax burden amount per resident (thousand KRW) in 2020	Number of Animal relinquishment in 2020	Human Population in 2021	Local tax burden amount per resident (thousand KRW) in 2021	Number of Animal relinquishment in 2021
Busan_8	306,003	199	760	301,987	215	669
Busan_9	105,164	233	515	105,192	241	436
Busan_10	175,095	250	336	174,806	280	322
Busan_11	207,144	216	417	203,536	249	366
Busan_12	396,438	333	659	386,785	358	480
Daegu Metropolitan City: 8 of 8 local governments						
Daegu_1	143,175	202	387	141,519	212	301
Daegu_2	544,926	243	592	536,989	261	525
Daegu_3	262,451	693	1047	263,162	748	909
Daegu_4	341,436	249	528	339,530	262	408
Daegu_5	437,008	205	463	430,912	221	415
Daegu_6	164,528	231	646	159,827	244	684
Daegu_7	417,097	291	1004	411,553	333	803
Daegu_8	74,791	598	345	80,199	653	362
Incheon Metropolitan City: 9 of 10 local governments						
Incheon_1	69,693	635	779	69,803	642	636
Incheon_2	295,696	174	762	288,856	187	513
Incheon_3	518,272	248	704	506,181	274	625
Incheon_4	61,486	326	149	58,999	334	122
Incheon_5	407,464	188	711	406,004	193	637
Incheon_6	486,765	198	1084	489,118	213	1222
Incheon_7	555,380	391	1004	589,013	428	969
Incheon_8	389,644	417	441	385,796	408	320
Incheon_9	143,633	1,027	860	152,931	1,019	817
Gwangju Metropolitan City: 5 of 5 local governments						
Gwangju_1	404,221	240	921	400,654	239	829
Gwangju_2	215,575	148	479	212,379	199	499
Gwangju_3	103,470	267	359	105,909	295	322
Gwangju_4	427,114	160	1202	424,707	182	1043
Gwangju_5	291,231	215	595	287,401	224	592
Daejeon Metropolitan City: 5 of 5 local governments						
Daejeon_1	175,046	277	493	172,746	290	332
Daejeon_2	222,222	190	597	219,751	201	439
Daejeon_3	473,365	196	799	470,374	213	470
Daejeon_4	351,277	321	647	356,093	334	448
Daejeon_5	230,341	190	681	227,108	177	420

[1] The numbers of animal relinquishments of observed regions were obtained from the 2021 and 2022 annual reports on animal relinquishment by the Korean Animal Welfare Association.

Table S1. Observed Regions and Human Population, Local Tax Burden Amount Per Resident, and Numbers of Animal Relinquishments in 2020 and 2021 used in the Analysis[1] (continued).

Observed region	Human Population in 2020	Local tax burden amount per resident (thousand KRW) in 2020	Number of Animal relinquishment in 2020	Human Population in 2021	Local tax burden amount per resident (thousand KRW) in 2021	Number of Animal relinquishment in 2021
Ulsan Metropolitan City: 25 of 25 local governments						
Ulsan_1	313,819	351	931	310,638	359	795
Ulsan_2	154,096	257	422	151,711	267	396
Ulsan_3	218,735	347	436	218,670	356	325
Ulsan_4	222,059	1,196	1123	221,512	1,081	986
Ulsan_5	212,883	203	422	208,132	218	424
Sejong Self-Governing City: 1 of 1 local governments						
Sejong	371,895	1,970	330	383,591	2,067	537
Gyeonggi-Do: 24 of 31 local governments						
Gyeonggi_1	62,264	884	287	62,150	933	238
Gyeonggi_2	1,079,353	565	1369	1,076,535	568	1217
Gyeonggi_3	292,893	602	331	287,945	668	330
Gyeonggi_4	387,289	806	1109	391,462	803	1045
Gyeonggi_5	191,948	564	152	188,701	570	175
Gyeonggi_6	268,535	625	389	266,213	664	178
Gyeonggi_7	733,798	543	1936	737,353	563	1701
Gyeonggi_8	93,592	494	241	91,546	505	213
Gyeonggi_9	806,067	517	1172	790,128	560	978
Gyeonggi_10	930,948	1,160	978	922,518	1,215	856
Gyeonggi_11	1,183,714	746	1081	1,190,964	755	919
Gyeonggi_12	512,030	733	794	512,912	710	852
Gyeonggi_13	652,726	710	1621	641,660	714	1332
Gyeonggi_14	189,534	983	1093	188,842	1,065	936
Gyeonggi_15	547,178	706	391	548,228	747	261
Gyeonggi_16	236,368	730	702	243,432	730	642
Gyeonggi_17	1,077,508	838	931	1,074,971	866	939
Gyeonggi_18	163,356	768	163	160,221	725	135
Gyeonggi_19	463,661	440	466	463,724	432	400
Gyeonggi_20	223,177	1,362	922	222,721	1,283	822
Gyeonggi_21	483,245	723	766	495,315	702	777
Gyeonggi_22	564,288	1,055	2938	578,529	1,069	2121
Gyeonggi_23	320,087	764	580	326,059	920	602
Gyeonggi_24	887,015	1,375	2224	910,814	1,257	1796

[1] The numbers of animal relinquishments of observed regions were obtained from the 2021 and 2022 annual reports on animal relinquishment by the Korean Animal Welfare Association.

Table S1. Observed Regions and Human Population, Local Tax Burden Amount Per Resident, and Numbers of Animal Relinquishments in 2020 and 2021 used in the Analysis[1] (continued).

Observed region	Human Population in 2020	Local tax burden amount per resident (thousand KRW) in 2020	Number of Animal relinquishment in 2020	Human Population in 2021	Local tax burden amount per resident (thousand KRW) in 2021	Number of Animal relinquishment in 2021
Gangwon-Do: 4 of 18 local governments						
Gangwon_1	212,965	591	558	211,381	567	689
Gangwon_2	82,791	538	422	82,806	601	356
Gangwon_3	357,757	507	1,041	360,807	507	874
Gangwon_4	284,594	658	595	286,664	665	512
Chungcheongbuk-Do: 5 of 11 local governments						
Chungcheongbuk_1	45,773	535	225	44,956	563	178
Chungcheongbuk_2	50,093	667	377	49,520	676	446
Chungcheongbuk_3	92,197	1115	406	92,058	1126	329
Chungcheongbuk_4	848,482	651	1742	849,573	651	1553
Chungcheongbuk_5	209,358	629	569	208,277	641	409
Chungcheongnam-Do: 6 of 15 local governments						
Chungcheongnam_1	114,483	487	746	112,617	574	641
Chungcheongnam_2	176,645	1,024	754	176,413	890	775
Chungcheongnam_3	324,580	1,018	1300	334,539	967	1115
Chungcheongnam_4	76,801	703	517	77,385	748	483
Chungcheongnam_5	658,486	732	1000	657,559	733	1219
Chungcheongnam_6	99,324	638	730	98,068	667	537
Jeollabuk-Do: 5 of 14 local governments						
Jeollabuk_1	265,304	665	1,659	262,467	630	1,225
Jeollabuk_2	80,913	656	466	81,455	698	512
Jeollabuk_3	91,142	959	453	92,422	935	354
Jeollabuk_4	278,113	561	1,478	273,697	563	1,621
Jeollabuk_5	657,269	575	2,196	651,495	583	2,160
Jeollanam-Do: 3 of 22 local governments						
Jeollanam_1	116,726	941	634	116,456	900	1018
Jeollanam_2	218,589	455	1289	216,939	483	1096
Jeollanam_3	281,436	544	558	278,737	516	648

[1] The numbers of animal relinquishments of observed regions were obtained from the 2021 and 2022 annual reports on animal relinquishment by the Korean Animal Welfare Association.

Table S1. Observed Regions and Human Population, Local Tax Burden Amount Per Resident, and Numbers of Animal Relinquishments in 2020 and 2021 used in the Analysis[1] (continued).

Observed region	Human Population in 2020	Local tax burden amount per resident (thousand KRW) in 2020	Number of Animal relinquishment in 2020	Human Population in 2021	Local tax burden amount per resident (thousand KRW) in 2021	Number of Animal relinquishment in 2021
Gyeongsangbuk-Do: 4 of 23 local governments						
Gyeongsangbuk_1	268,369	695	1,019	267,725	681	931
Gyeongsangbuk_2	412,581	838	165	408,110	662	577
Gyeongsangbuk_3	140,239	651	504	139,324	640	424
Gyeongsangbuk_4	503,852	744	1,748	496,650	681	1,464
Gyeongsangnam-Do: 6 of 18 local governments						
Gyeongsangnam_1	241,216	593	1,424	236,662	612	1,225
Gyeongsangnam_2	537,673	731	1,749	535129	704	1,554
Gyeongsangnam_3	103,525	744	1,960	102945	800	2,008
Gyeongsangnam_4	354,726	838	366	353792	818	218
Gyeongsangnam_5	347,097	620	541	343782	629	662
Gyeongsangnam_6	1,032,741	730	1,884	1,021,487	750	1,389
Jeju Self-Governing Province: 1 of 1 local governments						
Jeju	676,759	2,327	6,303	678,159	2,257	5,235

[1] The numbers of animal relinquishments of observed regions were obtained from the 2021 and 2022 annual reports on animal relinquishment by the Korean Animal Welfare Association.
